# Pan4Draft: A Computational Tool to Improve the Accuracy of Pan-Genomic Analysis Using Draft Genomes

**DOI:** 10.1038/s41598-018-27800-8

**Published:** 2018-06-25

**Authors:** Allan Veras, Fabricio Araujo, Kenny Pinheiro, Luis Guimarães, Vasco Azevedo, Siomar Soares, Artur da Costa da Silva, Rommel Ramos

**Affiliations:** 1 Institute of Biological Sciences, Federal University of Pará, Belém, Brazil; 2 Institute of Biological Sciences, Federal University of Minas Gerais, Belo Horizonte, Brazil; 3 Institute of Biological Sciences, Federal University of Triângulo Mineiro, Uberaba, Brazil

## Abstract

High-throughput sequencing technologies are a milestone in molecular biology for facilitating great advances in genomics by enabling the deposit of large volumes of biological data to public databases. The availability of such data has made possible the comparative genomic analysis through pipelines, using the entire gene repertoire of genomes. However, a large number of unfinished genomes exist in public databases; their number is approximately 16-fold higher than the number of complete genomes, which creates bias during comparative analyses. Therefore, the present work proposes a new tool called Pan4Drafts, an automated pipeline for pan-genomic analysis of draft prokaryotic genomes to maximize the representation and accuracy of the gene repertoire of unfinished genomes by using reads from sequencing data. Pan4Draft allows to perform comparative analyses using different methodologies such as combining complete and draft genomes, using only draft genomes or only complete genomes. Pan4Draft is available at http://www.computationalbiology.ufpa.br/pan4drafts and the test dataset is available at https://sourceforge.net/projects/pan4drafts.

## Introduction

The acquisition of large volumes of data from high-throughput sequencing platforms has resulted in a tremendous increase in the number of genomes deposited in public databases^1^, enabling comparative analyses of genomes of different organisms^2^.

The primary questions that can be addressed by comparative genomics involve understanding the evolutionary processes of organisms, the relationship between conserved DNA sequences encoding important functional proteins, and identifying non-coding sequences and proteins with non-essential functions^3^.

Comparative genomics enables the comparison of structural features (including low complexity regions), identification of rearrangement events, evaluation of gene synteny, identification of orthologous and paralogous genes, analysis of conserved gene clusters, identification and analysis of gene fusion or division events between species, and the comparison of non-coding regions by identifying regulatory elements^4,5^.

Zhang and colleagues (2015) carried out comparative analyses to identify essential genes, which were subsequently evaluated for their presence in genomic islands^6^. Other studies use these approaches to identify gene rearrangements, gene duplication, and gene acquisition by lateral gene transfer^7^.

The use of complete genomes is recommended for evaluating the complete gene repertoire via comparative analysis. However, the number of unfinished genomes in public databases has increased drastically in recent years. According to the Genomes OnLine Database^8,9^, the number of complete and draft genomes deposited in public databases in 2016 reached 1,626 and 26,624, respectively. The increase in the number of genomes, completes or drafts, is more pronounced in bacteria^10^, due to the compact nature of their genomes and also due to their application in diverse fields, like biotechnological industries, agriculture, medicine and others.

Therefore, we present Pan4Drafts, a user friendly, graphic computational tool that aims to improve the accuracy and increase the number of genes represented in draft genomes, which affect positively in pan-genomic analyses for unfinished genomes. Besides the automatic integration of different tools, Pan4Draft allows the identification of products that were not previously identified in the assembly process; performs the identification and adjustments of frameshifts, reducing the number of broken products; automates all processing steps without the need of users’ interaction; standardizes files; and generates charts to facilitate the downstream analysis from users. Also, Pan4Drafts is able to use reads from different libraries (single-end, paired-end and mate-paired) to help closing gaps and adjusting frameshifts in draft genomes and it allows to perform different approaches such as combining complete and draft genomes, using only draft genomes or only complete genomes. However, we did not test how the reconstruction of pan-genomes is influenced by incomplete genomes. Pan-genomes created from draft genomes should thus still be treated with caution.

## Methods

### Pan4Draft pipeline

Pan4Draft receives the following input files: a contigs multifasta file generated during the preliminary process of assembly and gap closing and the reads used to assemble this file. Those reads will be mapped against the contigs multifasta to identify reads without hits, which will be used in a second *de novo* assembly process.

The contigs file resulting from this process is sent to the web-based RAST^11^ platform for annotation. During this step, there are automatic modules responsible for sending, managing the status, and downloading files at the end of the annotation process. At the end of the annotation and finalization of the EMBL-format file download, the CDSs are extracted and used in the gap closing process or extension of the contigs to reduce gap sizes.

The resulting multifasta file from the second assembly is mapped using BLAST^12^. The contigs without hits, and those that are at least 200 bp long are added to the end of the input file. The file with the added contigs created after gap closing is redirected to the RAST platform for annotation. Finally, the generated EMBL file is submitted to the module for identification of possible frameshifts.

**Figure 1:**
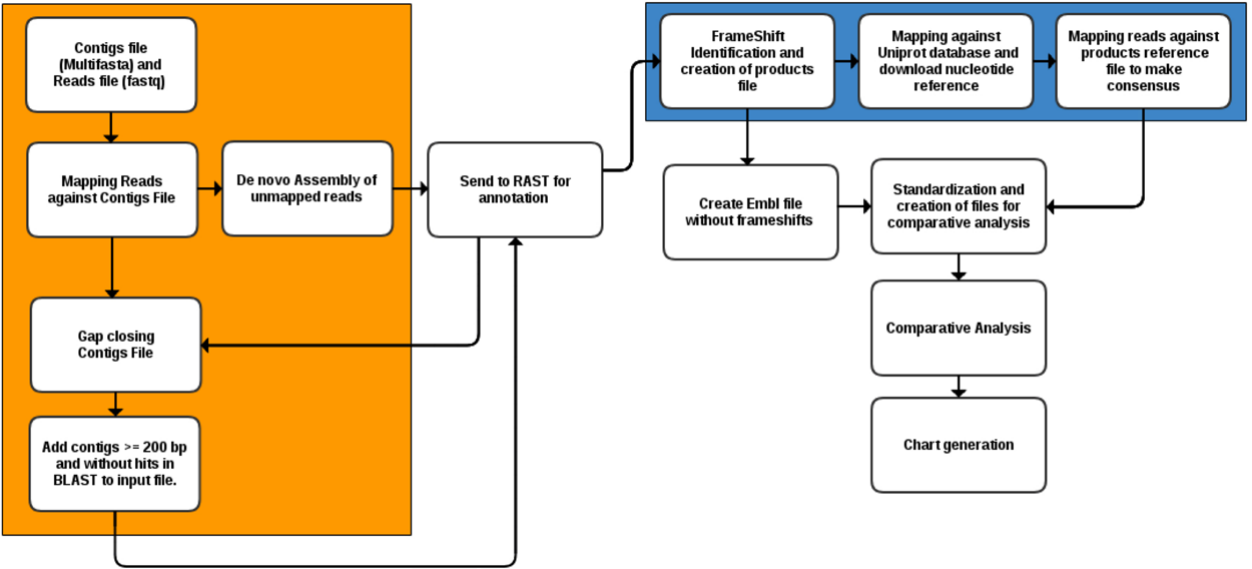
Pan4Draft pipeline. Processes highlighted in orange consist of the addition of gene content, which may have been left out of the contigs file due to assembly artifacts. Processes highlighted in blue are frameshift adjustment steps.

The file with the products identified as possible frameshifts is analyzed and adjusted, when possible, generating a new consensus from this adjustment. Both this consensus and the previously created EMBL file without frameshifts are used as input for the creation and standardization of comparative analysis files. PGAP^13^ is used for comparative analysis, and the resulting files are directed to R to generate charts. All steps of Pan4Draft are summarized in Fig. 1 and detailed in the next subsections.

### Mapping

Bowtie2 v.2.3.4.1^14^ was used to map the sequencing reads against the input file. During this process, a fastq file is created containing the unmapped reads. The default values of minins, maxins, mismatches in seed alignment (-N), and the length of seed substrings (-L) are 0, 500, 0, and 22, respectively.

### *De novo* assembly

Spades v.3.9.0^15^ was used for the de novo assembly of reads that were not mapped against the input file using the following parameters: -t 8, –careful, and automatic definition of the k-mer value. The parameter values can be customized by the user in the graphic interface.

### Mapping contigs against the consensus sequence

BLAST was used to map the contigs produced in the *de novo* assembly against the input file with an e-value threshold of 1E-05 and output in tabular format (-m 8) using 10 threads (-a 10). After mapping with BLAST, unmapped contigs that were at least 200 bp long were added at the end of the input file.

### Standardization of annotation

This step was performed using the web-based RAST platform. The RAST batch interface was used for data submission, status management, and downloading files in EMBL format.

### Identification and adjustment of possible frameshifts

The identification of possible frameshifts was based on an analysis of consecutive CDSs with the same annotation value. In this step, one EMBL file containing the products without frameshifts and one fasta file with the products identified as possibly frameshifts were created.

The file with possible frameshifts is used as input in BLAST in order to search the UniProt Database^16^ and obtain the unique sequence identifier that is subsequently used to download the reference in nucleotide format. Bowtie2 and SAMtools^17^ through mpileup are used together for mapping reads against reference files, which is followed by the generation of a new consensus from this result.

### BLAST and references download

The products identified as containing possible frameshifts were used as queries to perform a BLAST search against UniProtKB Database to obtain the unique ID mapping identifier that was later used to download nucleotide references from the European Nucleotide Archive.

### Consensus generation

Bowtie2 software was used to map the raw data against the downloaded product references. This mapping was submitted to SAMtools using the mpileup pipeline to create the consensus sequence of these products. The gene completeness (expressed as a percentage) input by the user was used to define the products added to the analysis.

### Standardization of archives for comparative analysis

The standardization module was used to format input files for comparative analysis. Files with the extensions nuc, pep, and function that contained the nucleotide sequence of the gene products, amino acid sequence, and information about the products predicted in the standardization of annotation step, respectively, were generated^13^.

### Comparative analysis

PGAP software is integrated to Pan4Draft and it was used for comparative analysis due to its simple and straightforward usage. The user must input the following parameters: –strains, –cluster, –thread, – value, –identity, and –coverage. If the user wants to use other software for pangenomic analysis, such as Roary^18^, panX^19^ and OrthoMCL^20^, it is possible to select the generation of a fasta file resulted from all the steps before the comparative analysis.

### Chart generation

The R statistical computing environment was used to generate charts for the analysis of results. The packages used to generate the graphics include plotrix, minpack.lm, and ctv. Charts were generated to show the number of unique genes, the number of shared cluster orthologs, and phylogenetic trees with two pie charts showing the number of shared orthologs (https://www.r-project.org/).

### Programming language and database

JAVA (http://www.oracle.com/) was the programming language used in the development of the computational tool with the graphical interface developed using the Swing graphic library (http://www.oracle.com/). The database used to control the status of all pipeline steps was SQLite version 3 (https://www.sqlite.org/).

### Pipeline validation

A pan-genomic analysis was performed using both draft and complete genomes. Seven strains of complete genomes of *Escherichia coli* were used as control. Six of then were selected to act as draft genomes and one as complete genomes. The drafts genomes are: *E. coli* P12b, *E. coli* strain RR1, *E. coli* K-12 str. *GM4792* Lac+, *E. coli* 042, *E. coli* KLY, *E. coli* O25b:H4-ST131, and the complete genome is *E. coli* IAI39, and the complete sequencing data of these organisms are available in the NCBI database under the accession numbers SRX1012260, SRX1021885, SRX1295865, ERX002221, SRX610250, SRX321704, and SRX1134025, respectively. It is important to mention that availability of complete genomes and the raw sequencing data that were used to assembly these genomes are very scarce. For this reason, we were able to validate our tool using only 7 genomes.

Draft genomes were generated from sequencing data of the complete genomes after an assembly process using the CLC Genomics Workbench software version 8.0 (www.qiagenbioinformatics.com) and subsequent curation in the Lasergene SeqMan Pro tool (https://www.dnastar.com), both using default parameters. The number of contigs produced in this process was 175, 164, 144, 273, 98, and 2985 in the SRR2000272, SRR2537294, SRR2014554, ERR007646, SRR1424625, and SRR933487 strains, respectively. SRX1134025 is a complete genome so its assembly produced 1 contig. Complete genomes and draft genomes were annotated in the RAST platform.

We perform two analyses: one using the core genome and the other using the accessory genome. In both cases we compared the results of the pan-genomic analysis before and after the use of Pan4Draft. Pan-genomic analysis of the controls genomes and the genomes before the use of Pan4Draft was performed by PanWeb^21^ and the pan-genomic analysis in Pan4Draft was performed by PGAP, as it was mentioned in earlier.

To define the parameters for the comparative analysis, we used the software Gegenees^22^, which performs an identity assessment between organisms, using the following parameter values: e-value = 0.00001, identity = 0.7 and coverage = 0.8. In the case of draft genomes, the value used for gene completeness was 70% therefore, during the process of mapping the reads against possible frameshift product references, only those with at least 70% of their content represented were considered.

## Results

### Pan4Drafts software

Initially, the user is presented with a Project Manager where it is possible to load previous projects or create new ones. When creating a new project, users need to define parameters for the analysis in three steps. All steps have a user friendly, visual interface to facilitate usage of our tool. The first step requires the entry of a username and password previously created in the web-based RAST platform, which is used for the annotation process. The second step involves adding information about the organism: domain, taxonomy id, and location of the multifasta input file containing the contigs and the raw sequencing read files with their respective library type (single-end or paired-end). If the reads are paired, they should be reported in order along with the read orientation information (forward/forward; forward/reverse). The user can follow the entry of information through the system interface. In the final step, one must add the parameters of the assembler, the aligner and and the pan-genomic analysis tool. More detailed information regarding the complete parameters of Pan4Draft is available in the user’s manual.

To increase the representation of the gene repertoire of draft genomes analyzed by the software prior to pan-genomic analysis, the following steps are performed: genome submission to the annotation environment, downloading of results, annotation process status management, identification of possible frameshifts and download of its identified products’ references. Before starting the analysis process with Pan4Drafts it is advisable that the user has carried out a process of refinement in the strains. These previous steps increase the quality of the strains and may impact on the improvement of the results obtained.

### Analysis of similarity, frameshifts, sinteny and pan-genome

**Figure 2:**
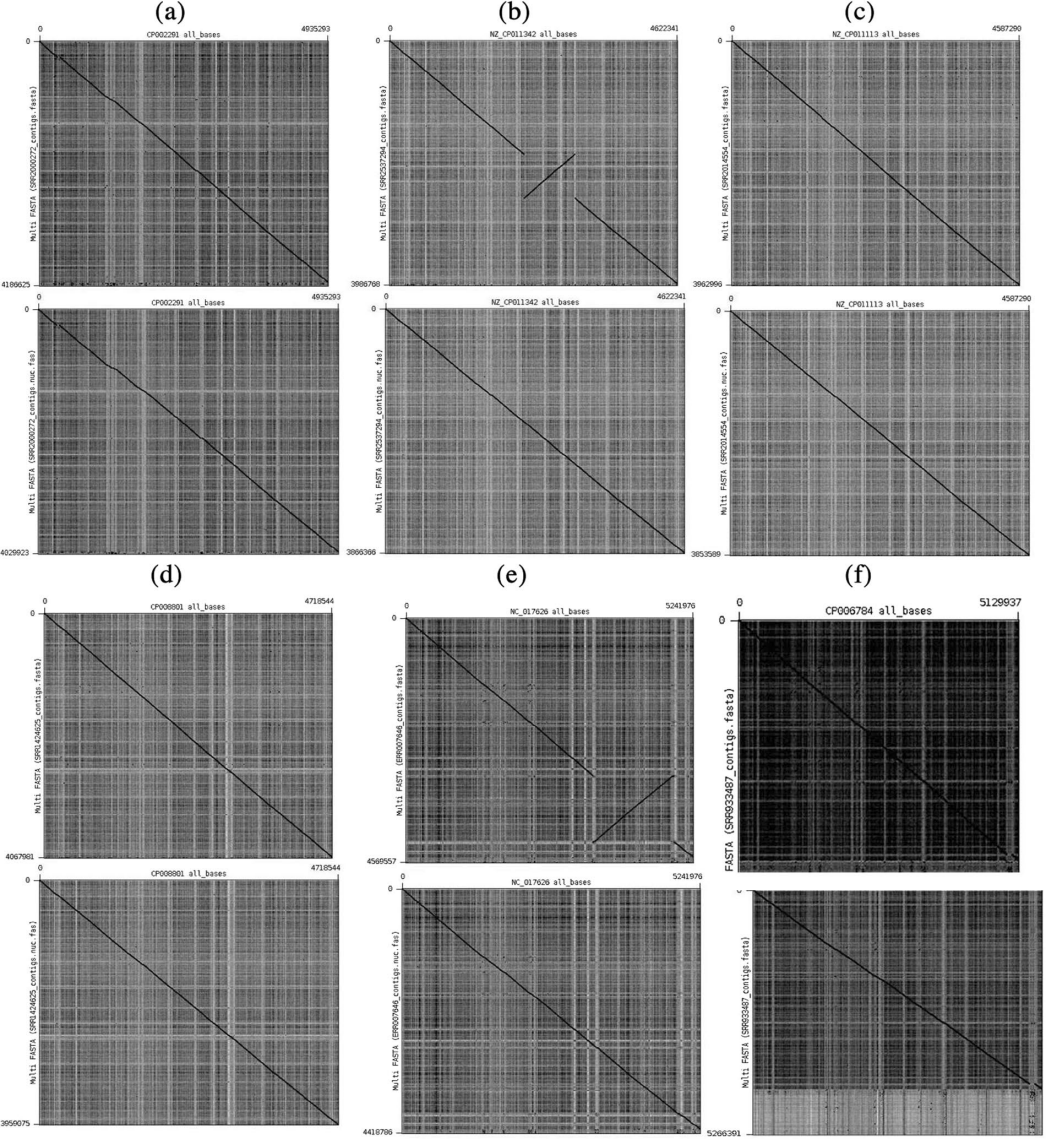
Synteny analysis of the genomes and its reference. For each letter, the top side of the figure represents the genomes before using Pan4Draft and the bottom side represents the genomes after using Pan4Draft. The X axe represents the reference and the Y axe represents the ordered multifasta file. (**a**) the synteny analysis of E. coli P12b (SRA SRX1012260, Run SRR2000272. (**b**) synteny analysis of E. coli K-12 str. GM4792 Lac+ (SRA SRX1295865, RUN SRR2537294). (**c**) E. coli RR1 (SRA SRX1021885, RUN SRR2014554). (**d**) E. coli KLY (SRA SRX610250, RUN SRR1424625). (**e**) E. coli 042 (SRA ERX002221, RUN ERR007646). (**f**) E. coli 042 (SRA ERX002221, RUN ERR007646).

**Figure 3:**
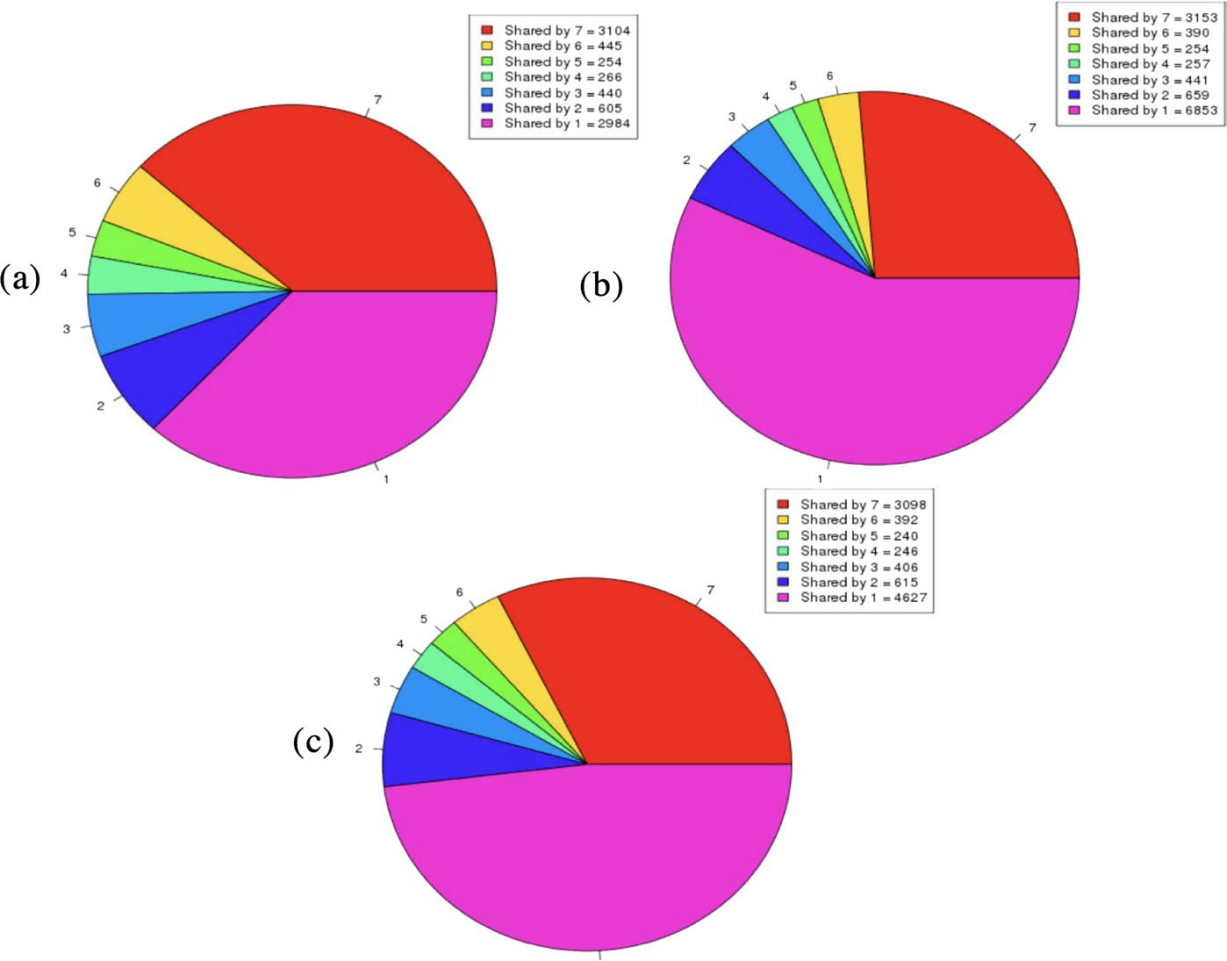
Orthologs cluster analysis of the pan-genome shows that the clusters are more similar between the complete genome and the genome after using Pan4Draft. (**a**) represents the orthologs cluster of the complete genomes (**b**) represents the orthologs cluster of the genome before using Pan4Draft and (**c**) represents the orthologs cluster after using Pan4Draft.

To determine the degree of accuracy of Pan4Draft, we assessed the similarity of products (Table 1), the number of frameshifts (Table 2), the sinteny (Fig. 2) and the ortholog clusters (Fig. 3) before and after the use of Pan4Draft.

**Table 1:** Evaluation of accuracy between draft and complete genomes for different strains before and after using Pan4Draft (core and accessory genomes).

Organism	Before Pan4Draft				After Pan4Draft		
	Total products from complete genome	Total products found	Products with 100% similarity (core)	Products with 100% similarity (accessory)	Total products found	Products with 100% similarity (core)	Products with 100% similarity (accessory)
*E. coli* P12b (SRA SRX1012260, Run SRR2000272)	4920	4664	2937 (62.97%)	800 (17.15%)	4437	2895 (65.25%)	760 (17.13%)
*E. coli* K-12 str. GM4792 Lac + (SRA SRX1295865, RUN SRR2537294)	4396	4356	3109 (71.37%)	974 (22.36%)	4188	3041 (72.61%)	911 (21.75%)
*E. coli* RR1 (SRA SRX1021885, RUN SRR2014554)	4369	4339	3106 (71.58%)	962 (22.17%)	4186	3052 (72.91%)	896 (21.40%)
*E. coli* KLY (SRA SRX610250, RUN SRR1424625)	4501	4461	3092 (69.31%)	1009 (22.62%)	4330	3017 (69.68%)	947 (21.87%)
*E. coli* 042 (SRA ERX002221, RUN ERR007646)	5130	5150	3090 (60.00%)	931 (18.08%)	4927	3029 (61.48%)	900 (18.27%)
*E. coli* O25b:H4-ST131 (SRA SRX321704, RUN SRR933487)	5007	8882	3036 (34.18%)	871 (9.81%)	6684	3010 (45.03%)	861 (12.88%)
*E. coli* IAI39 (SRA SRX1134025, RUN SRR2146161)	5032	5032	3138 (62.36%)	965 (19.18%)	5032	3082 (61.25%)	962 (19.12)

**Table 2:** Analysis of amount frameshifts found.

Organism	Before Pipeline	After Pipeline
*E. coli* P12b (SRA SRX1012260, Run SRR2000272)	349	227
*E. coli* K-12 str. GM4792 Lac + (SRA SRX1295865, RUN SRR2537294)	227	194
*E. coli* RR1 (SRA SRX1021885, RUN SRR2014554)	273	174
*E. coli* KLY (SRA SRX610250, RUN SRR1424625)	287	177
*E. coli* 042 (SRA ERX002221, RUN ERR007646)	374	274
*E. coli* O25b:H4-ST131 (SRA SRX321704, RUN SRR933487)	998	855
*E. coli* IAI39 (SRA SRX1134025, RUN SRR2146161)	367	367

To perform the synteny analysis, the multifasta file of each organism was ordered based on its reference using the Mauve software^23^. The results were used as input for Gepard software which generated the charts for genic conversion (Fig. 2). In some cases, there is no significant changes in the charts before and after using Pan4Draft. However, in other cases, as seen in Fig. 2b,e, there is a inversion of the synteny before using Pan4Draft that was corrected after it was used.

After the analysis of PGAP, we evaluated the ortholog clusters generated by PGAP, as seen in Fig. 3. Its possible to observe that the ortholog clusters after using Pan4Draft is more similar to the complete genome than the ortholog clusters before using Pan4Draft.

**Figure 4:**
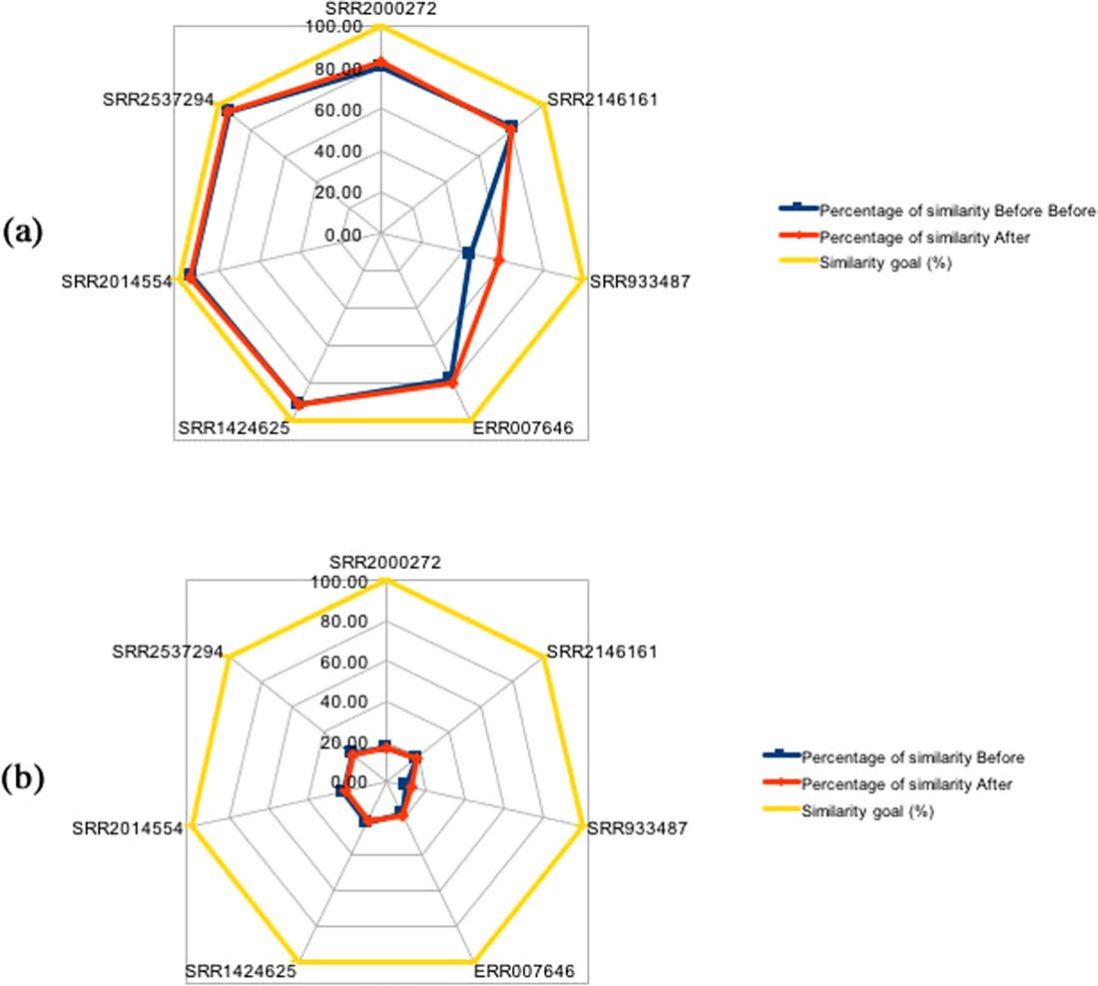
Plot of similarity values observed before and after the use of the Pan4Draft for the analysis using the core (**a**) and accessory genome (**b**) the controls (complete genomes) are shown in yellow.

All products present in the core genome and accessory genome were used to determine the degree of similarity and compare the results. For this purpose, a multifasta containing the products of each result was created. The Fig. 4a,b show the degree of similarity between the analyzes performed before and after the use of Pan4Draft, for the analysis using only the core genome and the analysis with only the accessory genome, respectively. It is observed that when the use of the Pan4Draft occurred, the degree of similarity reached is close to the control value (complete genomes). So, from the data of Table 1 and Fig. 4, it is possible to observe that, after running Pan4Drafts, the total of products is lower. However, it is also possible to observe that the number of frameshifts is also lower. So, when we compare the total of products, the number of products with 100% of similarity and the number of frameshifts, it is clear that the data generated after using Pan4Drafts is more accurate.

Finally, in order to characterize the pan-genome as open or closed, we must check the alfa value of the analysis^24^. The results obtained for the analysis, based on the median were: 0.7288, 0.4396 and 0.5728, for the analysis to complete genome, draft genome before and after using Pan4Draft, respectively.

### Output files

After finishing the pipeline, the following graphs are plotted (Supplementary Material): Figs 2–4 Bar chart showing the number of unique genes identified in each organism; Supplementary Figures 5–7 bloxplot exhibit the pangenome and central genome (the bloxplots are identified with the blue and red colors respectively). This graph also shows the curve of the Heap Law adjusted by the mean values (yellow curve) and median (green curve), the alpha value used to classify the open or closed pangenome is shown in the same graph; Supplementary Figures 8–10 are phylogenetic trees based on the UPGMA algorithm, Supplementary Figure 8 is constructed based on the gene distance matrix for clusters of major genes, Supplementary Figure 9 is based on the indel variations in the nucleus-gene clusters; and Supplementary Figure 10 phylogenetic tree based on the ML algorithm displays the evolution analysis of the species based on indel variations in the nucleus-gene clusters.

### Run time evaluation

The processing time in seconds involved in a local execution (Intel Core i7-4510U 2.00 GHz, 16GB of RAM memory) of the pipeline are shown in Table 3 below.

**Table 3:** Processing time for the local execution of the steps in seconds: Step 1-Bowtie mapping against input file, Step 2-De novo assembly using Velvet, Step 3-BLAST contigs, Step 4-Adding contigs, Step 8-Frameshift identification, Step 11-Standardization of archives, Step 12-Comparative analysis. Step 9 (remote Uniprot blast) and step 10 (consensus generation of products with frameshifts based on reads) are run in remote environments so their processing time are not shown.

Organism	Step 1	Step 2	Step 3	Step 4	Total Contigs	Step 8	Step 11	Step 12
*E. coli* P12b (SRA SRX1012260, Run SRR2000272)	645.47	2563.29	71.69	1.29	5	0. 35	16.23	2033.01
*E. coli* K-12 str. GM4792 Lac + (SRA SRX1295865, RUN SRR2537294)	1131.82	1175.06	71.25	1.91	0	0.32	23.55	2033.01
*E. coli* RR1 (SRA SRX1021885, RUN SRR2014554)	1046.82	1205.18	71.70	1.20	6	0.36	18.50	2033.01
*E. coli* KLY (SRA SRX610250, RUN SRR1424625)	735.57	1296.20	71.39	1.10	0	0. 37	25.71	2033.01
*E. coli* 042 (SRA ERX002221, RUN ERR007646)	582.22	1508.30	71.34	2.23	3	0.62	24.95	2033.01
*E. coli* O25b:H4-ST131 (SRA SRX321704, RUN SRR933487)	389.06	2166.90	71.40	2.54	229	0. 59	28.15	2033.01
*E. coli* IAI39 (SRA SRX1134025, RUN SRR2146161)	—	—	—	—	—	—	24.10	—

## Conclusion

As standard, comparative analysis is performed between organisms that have their genome closed. They are characterized by having fully represented gene content, while the genomes in drafts have their contents partially represented which makes this type of analysis impracticable for these genomes. The development of Pan4Drafts made possible to perform the pan-genomic analysis using draft genomes by the identification and adjustment of frameshifts, reducing their quantity significantly, automating the processes without the need of user intervention and standardization of the files to be used in the comparative analysis. In addition, to provide the identification of products that were not previously represented in the assembly process, the results of this analysis are plotted in graphs that help the downstream analysis by the user.

## Electronic supplementary material

Detailed information of the experiment
