# Supplementary material for: Pan4Draft: A Computational Tool to Improve the Accuracy of Pan-Genomic Analysis Using Draft Genomes
Source: Sci Rep. 2018 Jun 25;8:9670. doi: 10.1038/s41598-018-27800-8 (PMC6018222; doi:10.1038/s41598-018-27800-8)
Supplement: Supplementary file 1 — Detailed information of the experiment [file 41598_2018_27800_MOESM1_ESM.pdf]

PAN4DRAFT: A COMPUTATIONAL TOOL TO IMPROVE THE ACCURACY OF PAN-GENOMIC ANALYSIS USING DRAFT GENOMES

Allan Veras[1,+], Fabricio Araujo[1,+], Kenny Pinheiro[1], Luis Guimarães[1], Vasco Azevedo[2], Siomar Soares[3], Artur da Costa da Silva[1], and Rommel Ramos[1,*]

[1]Institute of Biological Sciences, Federal University of Pará, Belém, Brazil
[2]Institute of Biological Sciences, Federal University of Minas Gerais, Belo Horizonte, Brazil
[3]Institute of Biological Sciences, Federal University of Triângulo Mineiro, Uberaba, Brazil
[*]rommelthiago@gmail.com
[+]these authors contributed equally to this work

ABSTRACT

High-throughput sequencing technologies are a milestone in molecular biology for facilitating great advances in genomics by enabling the deposit of large volumes of biological data to public databases. The availability of such data has made possible the comparative genomic analysis through pipelines, using the entire gene repertoire of genomes. However, a large number of unfinished genomes exist in public databases; their number is approximately 16-fold higher than the number of complete genomes, which creates bias during comparative analyses. Therefore, the present work proposes a new tool called Pan4Drafts, an automated pipeline for pan-genomic analysis of draft prokaryotic genomes to maximize the representation and accuracy of the gene repertoire of unfinished genomes by using reads from sequencing data. Pan4Draft allows to perform comparative analyses using different methodologies such as combining complete and draft genomes, using only draft genomes or only complete genomes. Pan4Draft is available at http://www.computationalbiology.ufpa.br/pan4drafts and the test dataset is available at https://sourceforge.net/projects/pan4drafts.

Table 1. Similarity analysis using the BLAST software considering the genes present in the central genome and accessory.

ORGANISM	BEFORE PIPELINE			AFTER PIPELINE			COMPLETE GENOMES	
	Total Products	Match 100%	Percentage of similarity Before	Total Products	Match 100%	Percentage of similarity After	Total products	Similarity goal (%)
SRR2000272	4664	3737	80.12	4437	3655	82.38	4920	100
SRR2537294	4356	4083	93.73	4188	3952	94.36	4396	100
SRR2014554	4339	4068	93.75	4186	3948	94.31	4369	100
SRR1424625	4461	4101	91.93	4330	3964	91.55	4501	100
ERR007646	5150	4021	78.08	4927	3929	79.74	5130	100
SRR933487	8882	3907	43.99	6684	3871	57.91	5007	100
SRR2146161	5032	4103	81.54	5032	4044	80.37	5032	100
		Average	80.45		Average	82.95		
		Mean of percentage difference Draft vs Complete	19.55		Mean of percentage difference Draft vs Complete	17.05		

Table 1. Similarity analysis using the BLAST software considering the genes present in the central genome.

ORGANISM	BEFORE PIPELINE			AFTER PIPELINE			COMPLETE GENOMES	
	Total Products	Match 100%	Percentage of similarity Before	Total Products	Match 100%	Percentage of similarity After	Total products	Similarity goal (%)
SRR2000272	4664	2937	62.97	4437	2895	65.25	4920	100
SRR2537294	4356	3109	71.37	4188	3041	72.61	4396	100
SRR2014554	4339	3106	71.58	4186	3052	72.91	4369	100
SRR1424625	4461	3092	69.31	4330	3017	69.68	4501	100
ERR007646	5150	3090	60.00	4927	3029	61.48	5130	100
SRR933487	8882	3036	34.18	6684	3010	45.03	5007	100
SRR2146161	5032	3138	62.36	5032	3082	61.25	5032	100
		Average	61.68		Average	64.03		
		Mean of percentage difference Draft vs Complete	38.32		Mean of percentage difference Draft vs Complete	35.97		

Table 1. Similarity analysis using the BLAST software considering the genes present in the accessory genome.

ORGANISM	BEFORE PIPELINE			AFTER PIPELINE			COMPLETE GENOMES	
	Total Products	Match 100%	Percentage of similarity Before	Total Products	Match 100%	Percentage of similarity After	Total products	Similarity goal (%)
SRR2000272	4664	800	17.15	4437	760	17.13	4920	100
SRR2537294	4356	974	22.36	4188	911	21.75	4396	100
SRR2014554	4339	962	22.17	4186	896	21.40	4369	100
SRR1424625	4461	1009	22.62	4330	947	21.87	4501	100
ERR007646	5150	931	18.08	4927	900	18.27	5130	100
SRR933487	8882	871	9.81	6684	861	12.88	5007	100
SRR2146161	5032	965	19.18	5032	962	19.12	5032	100
		Average	18.77		Average	18.92		
		Mean of percentage difference Draft vs Complete	81.23		Mean of percentage difference Draft vs Complete	81.08		

Table 2. Analysis of amount frameshifts

ORGANISM	BEFORE PIPELINE	AFTER PIPELINE	COMPLETE GENOMES
SRR2000272	349	227	460
SRR2537294	279	194	285
SRR2014554	273	174	273
SRR1424625	287	177	289
ERR007646	374	274	385
SRR933487	998	855	424
SRR2146161	367	367	367

Figure 1. Analysis of similarity between: (A) genes present in the core genomes; (B) genes present in the core and accessory genomes; (B) genes present in accessory genomes.

(A)

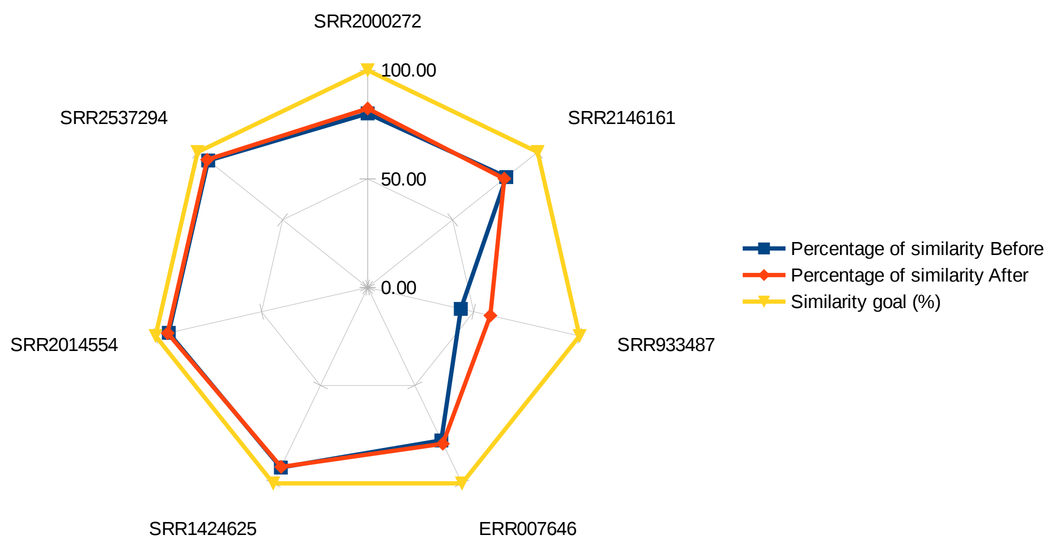

(B)

(C)

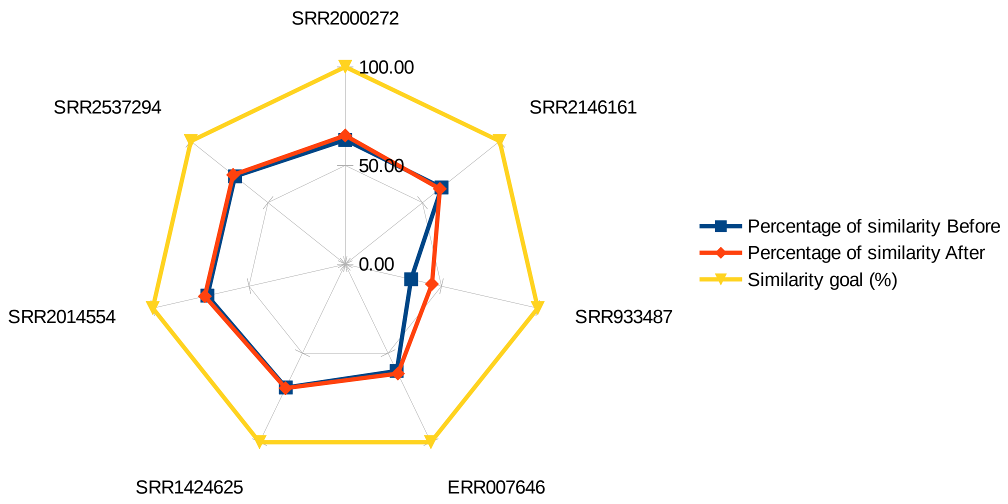

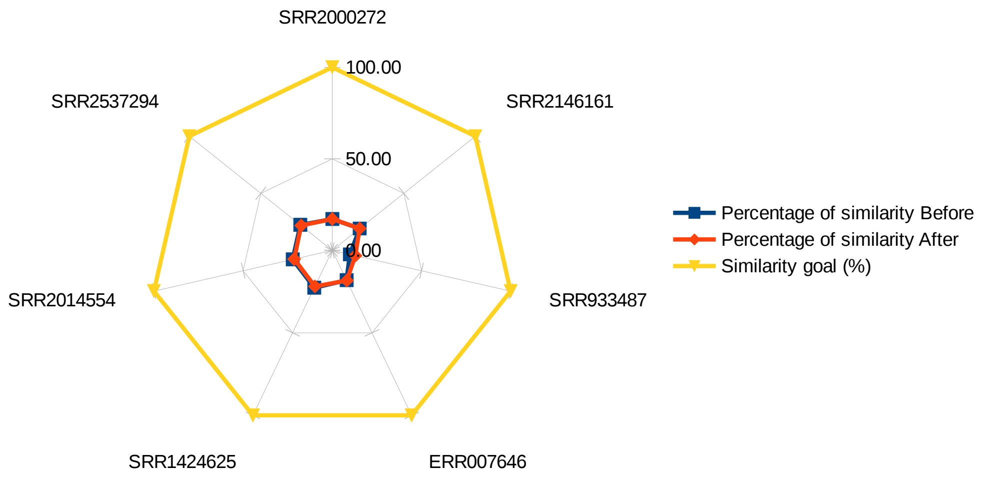

Figure 2. The result uniques for each strain for complete genome analysis.

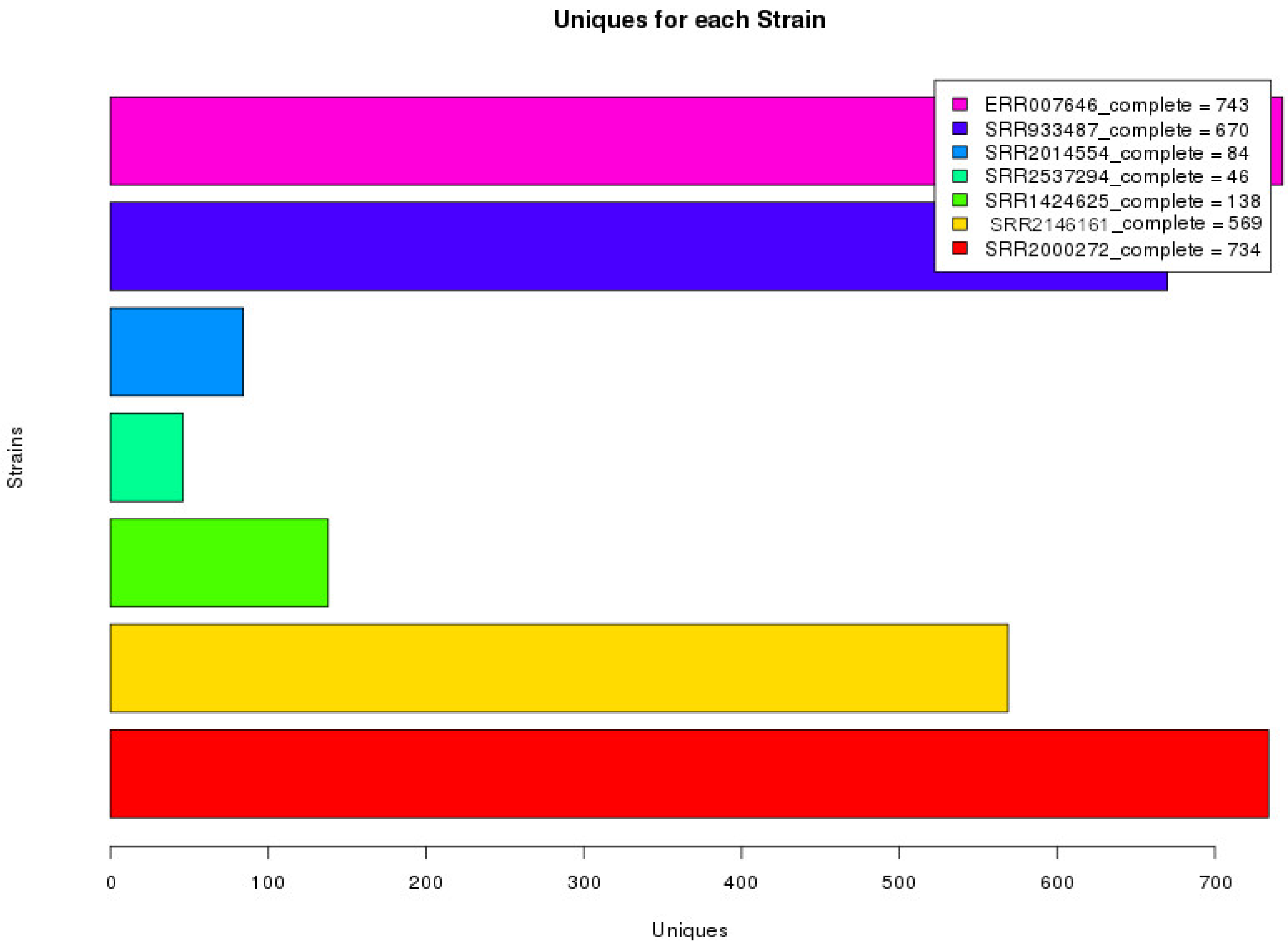

Figure 3. The result uniques for each strain for before pipeline.

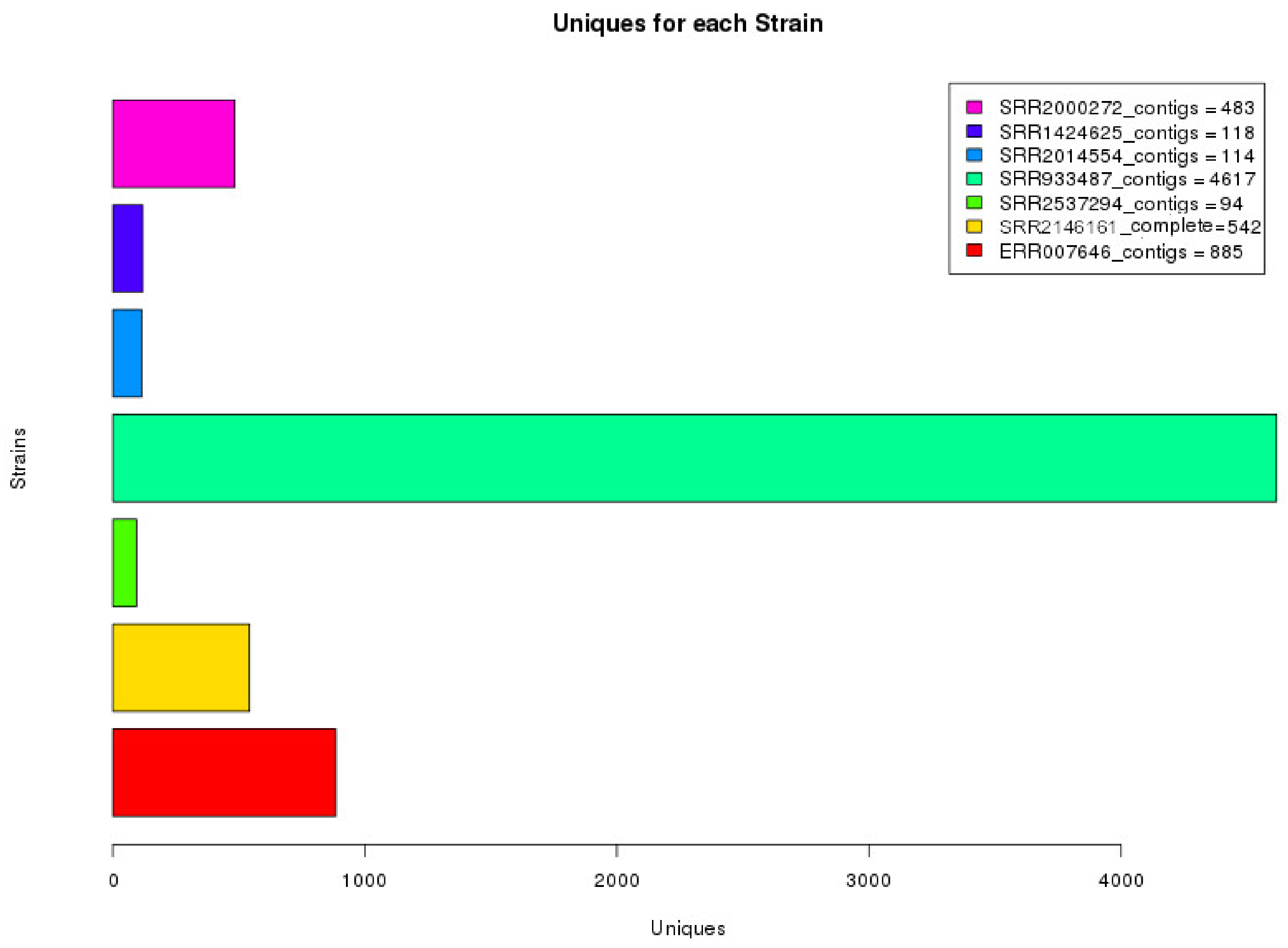

Figure 4. The result uniques for each strain for after pipeline.

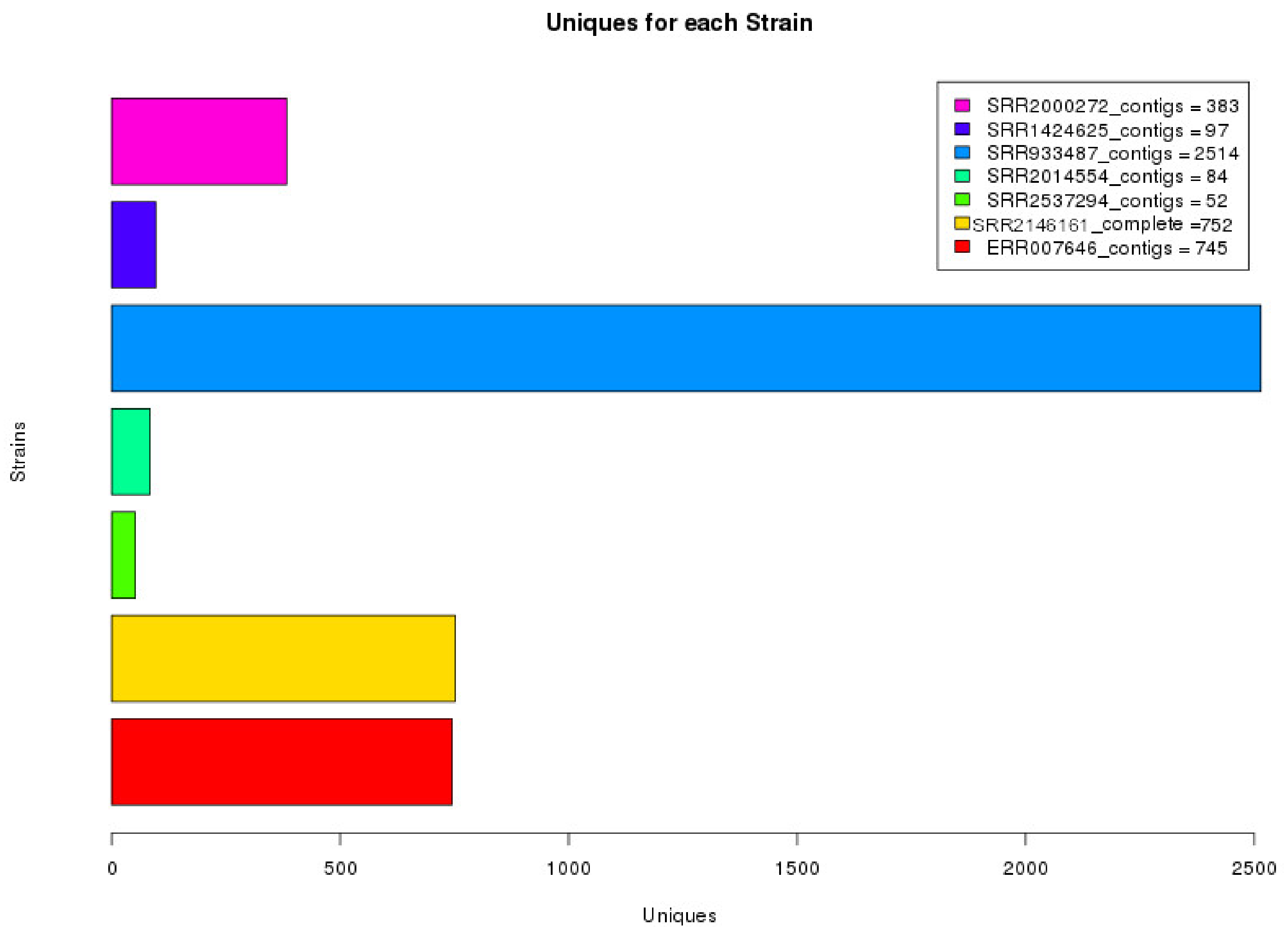

Figure 5. The result pangenome analysis complete genomes.

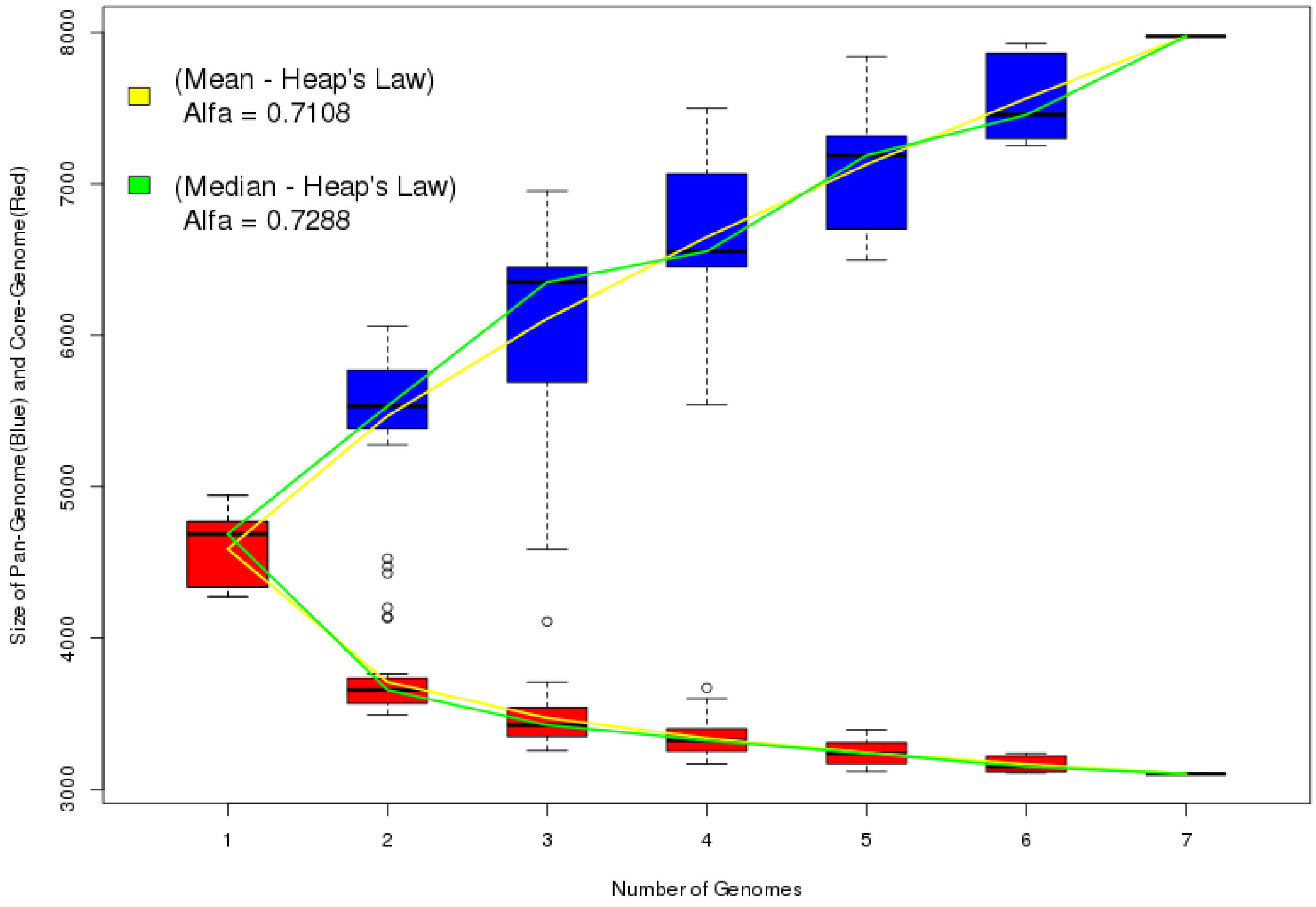

Figure 6. The result pangenome analysis before pipeline.

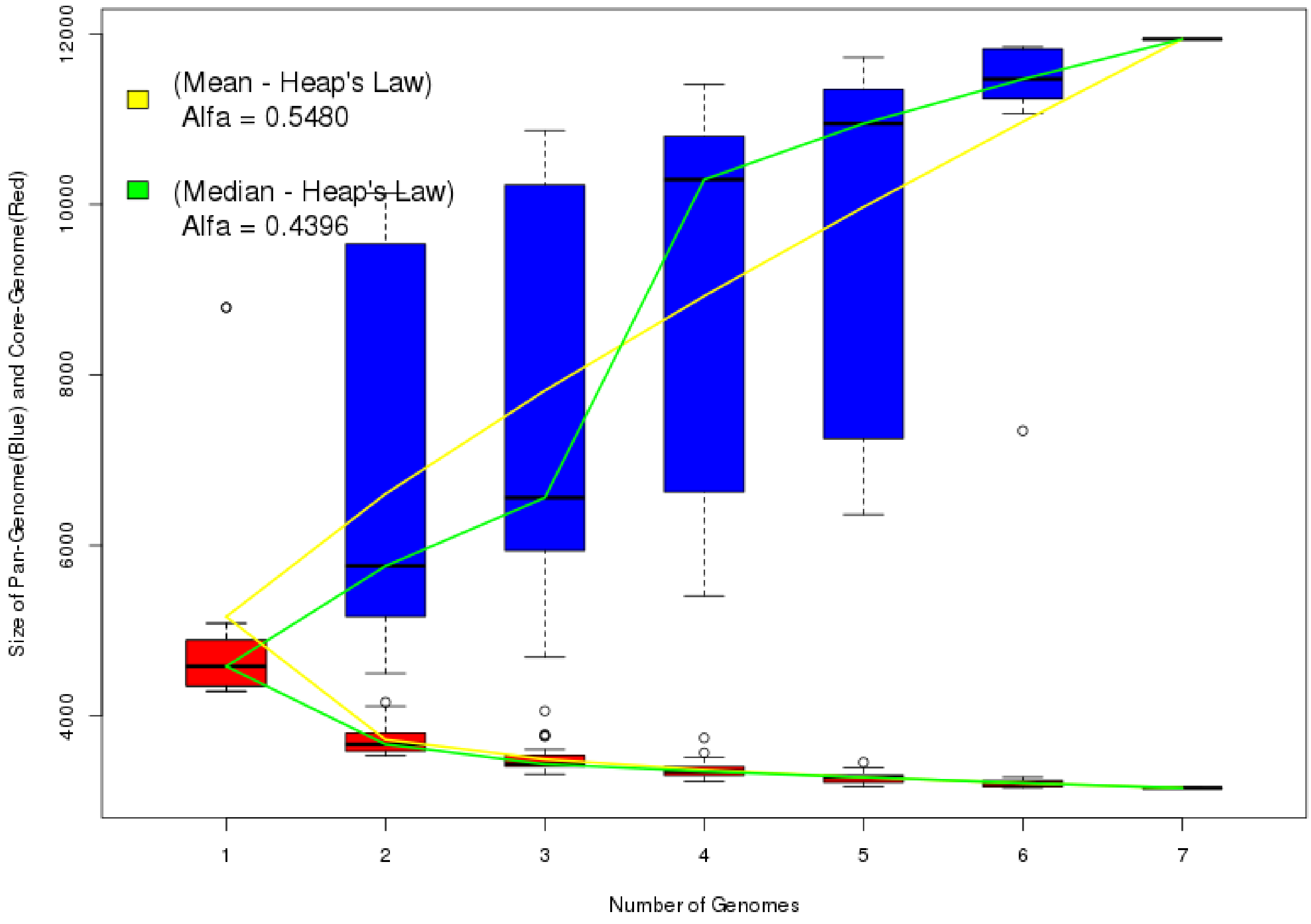

Figure 7. The result pangenome analysis after pipeline.

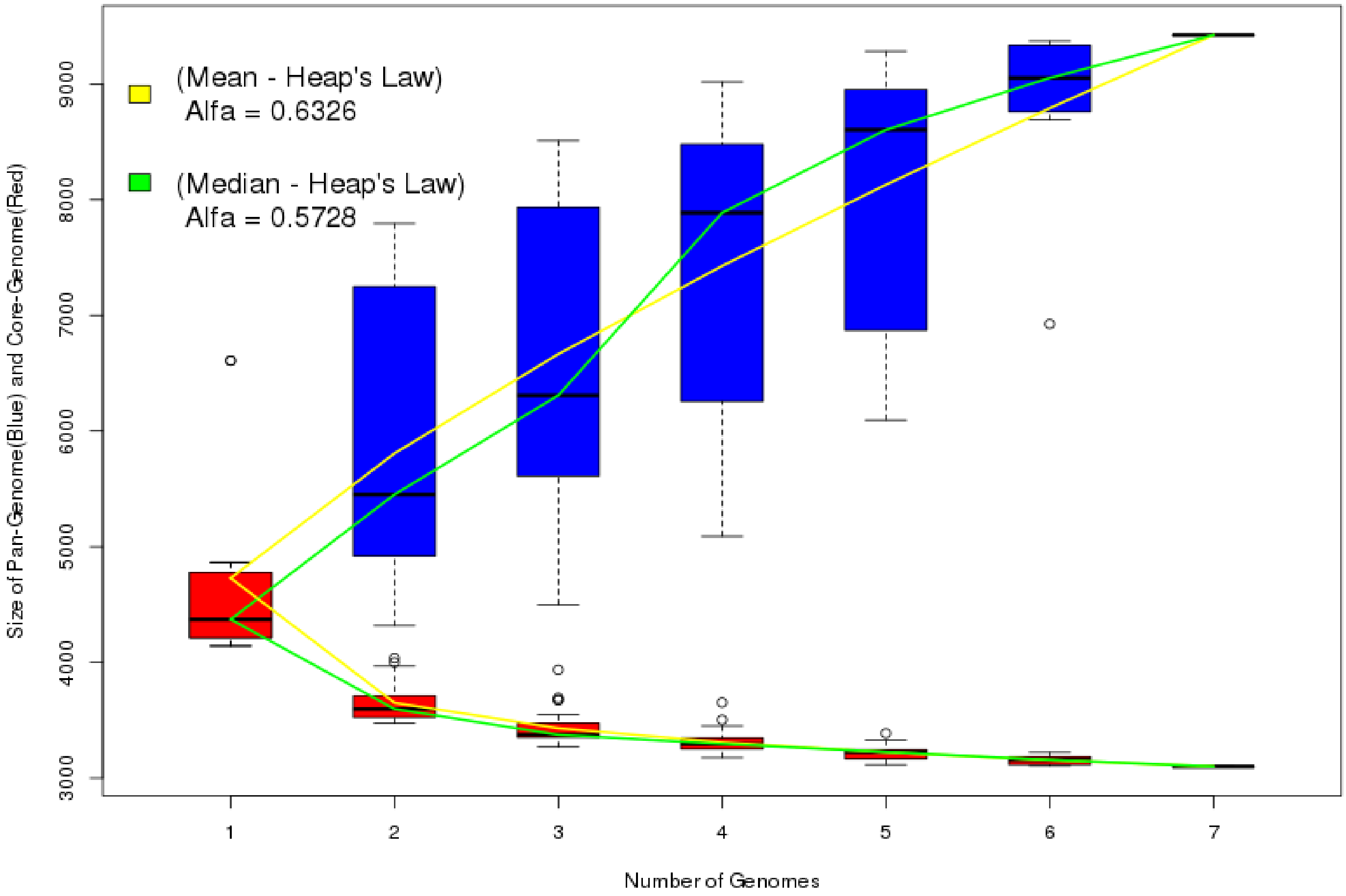

Figure 8. Phylogenetic tree based on the UPGMA algorithm, constructed based on the gene distance matrix for clusters of major genes.

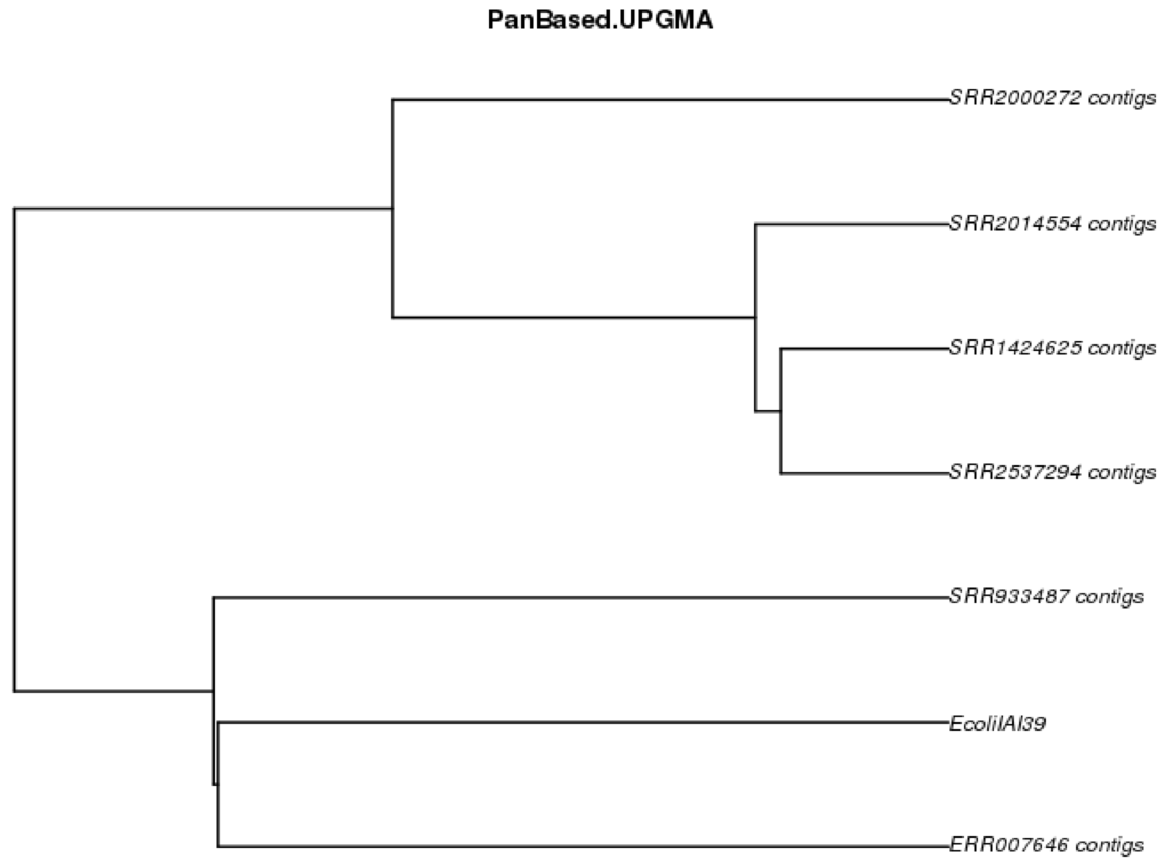

Figure 9. Phylogenetic tree based on the UPGMA algorithm, based on the indel variations in the nucleus-gene clusters

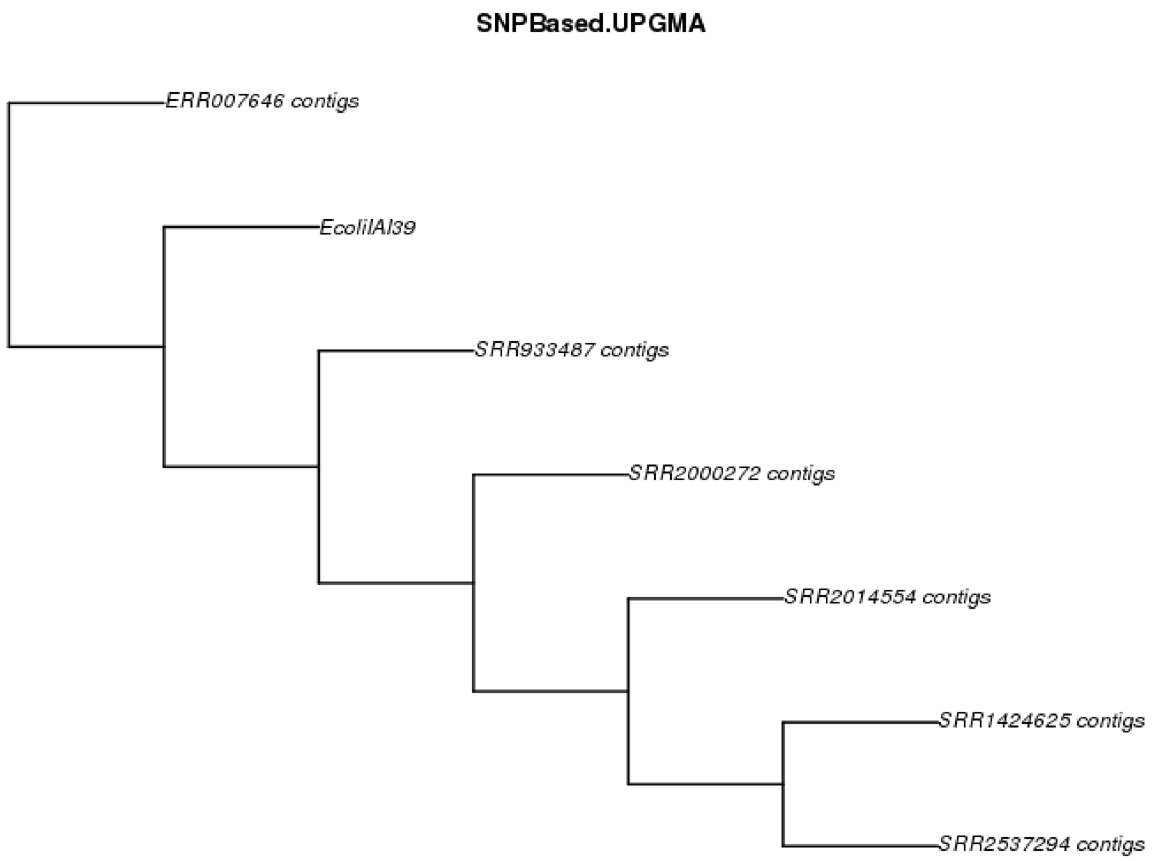

Figure 10. Phylogenetic tree based on the ML algorithm

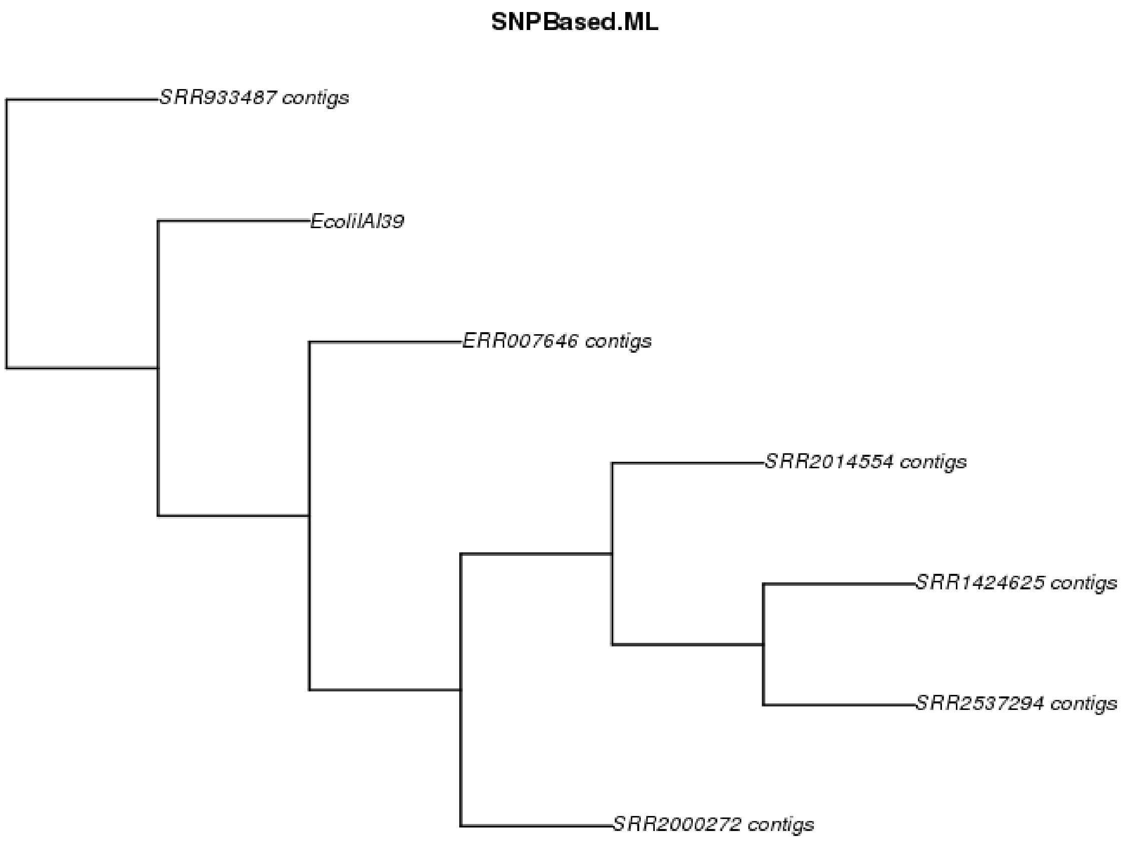

Figure 11. The results analysis with Gegenees Software.

Organism	1	2	3	4	5	6	7
1: ERR007646_complete	100	73	74	73	76	74	74
2: SRR1424625_complete	81	100	90	96	79	98	78
3: SRR2000272_complete	79	87	100	86	77	87	76
4: SRR2014554_complete	84	99	92	100	82	99	80
5: SRR2146161_complete	79	74	75	74	100	74	78
6: SRR2537294_complete	83	100	92	98	81	100	79
7: SRR933487_complete	76	72	72	71	76	72	100
